# Primary care registration of the mental health needs of patients treated at outpatient specialized services: results from a medium-sized city in Brazil

**DOI:** 10.1186/s12913-021-07127-3

**Published:** 2021-10-15

**Authors:** Carlos Alberto dos Santos Treichel, Ioannis Bakolis, Rosana Teresa Onocko-Campos

**Affiliations:** 1 Department of Collective Health, School of Medical Sciences, University of Campinas, St. Tessália Vieira de Camargo, 126, Cidade Universitária Zeferino Vaz, Campinas, SP 13083-887 Brazil; 2 Health Services and Population Research Department, Centre for Implementation Science, Institute of Psychiatry, Psychology and Neuroscience, King’s College London, London, UK; 3 Department of Biostatistics and Health Informatics, Institute of Psychiatry, Psychology and Neuroscience, King’s College London, London, UK

**Keywords:** Shared care, Mental Health, Community mental Health services, Primary care, Collaborative care

## Abstract

**Background:**

Although matrix support seeks to promote integrating Primary Care with specialized mental health services in Brazil, little is known about the quantitative impact of this strategy on sharing cases between different levels of care. The aim of this study was to investigate the prevalence and factors associated with Primary Care registration of the mental health needs of patients treated at outpatient specialized services in a medium-sized city in Brazil with recent implementation of matrix support.

**Methods:**

This is a document-based cross-sectional study conducted through an analysis of 1198 patients’ medical records. Crude and adjusted associations with the outcome were explored using logistic regression.

**Results:**

The prevalence of cases registered in Primary Care was 40% (*n* = 479). Evidence was found for associations between the outcome and the patients being over 30 years old, and referral by emergency or hospital services. There was conversely an inverse association between the outcome and status as a patient from the Outpatient Clinic or from the Psychosocial Care Center for psychoactive substance misuse.

**Conclusions:**

Even with the provision of mechanisms for network integration, such as matrix support, our results suggest that more groundwork is necessary to ensure that sharing cases between Primary Care and specialized services is effective.

## Background

Efforts have been made worldwide to integrate specialized mental health services and Primary Care in order to reduce the treatment gap and offer better mental health care [1, 2]. A recently conducted literature review [3] synthesized evidence that patients receiving treatment in the integrated models tend to have better outcomes compared with those receiving regular care.

Different integration models in high-income countries have been classified based on the nature and level of coordination between providers. At the first level, patients are referred to another location (coordinated care). At the second level, providers provide care at the same location, but do not share treatment plans (co-located care). At the highest integration level, providers are part of the same team with a common treatment plan, and the patient receives care within a single system (integrated care) [3, 4].

From a community point of view, the mental Care network in Brazil mainly consists of Primary Care, where it is expected that most mental health needs are identified and treated, in addition to Psychosocial Care Centers and Outpatient Clinics. The Psychosocial Care Centers can be divided into modalities I and II according to the size of the city (less than 70,000 inhabitants/more than 70,000 inhabitants, respectively), in addition to modalities III and IV (operating 24/7 in municipalities with more than 200,000 inhabitants/more than 500,000 inhabitants, respectively), and are the main service responsible for admitting individuals with serious and persistent mental disorders. The Psychosocial Care Centers can also be specialized in caring for people with needs arising from psychoactive substance misuse/abuse, or in child-juvenile populations.

Psychosocial Care Centers have a team minimally composed of a physician with a background in mental health, a nurse, three higher education professionals (e.g., psychologist, social worker and occupational therapist), and four high school level professionals (e.g., nursing technician, administrative technician, educational technician and craftsman). Their work consists in offering comprehensive clinical care and social rehabilitation for patients and their families.

In turn, Outpatient Clinics are responsible for receiving patients with mental health needs who do not meet the criteria for admission to Psychosocial Care Centers, but who could not be managed in Primary Care because they demand specialized knowledge. These services generally have specialists from different medical disciplines, and patients are assisted by psychiatrists and/or psychologists depending on their needs.

Attempts to integrate specialized mental health services and Primary Care have taken place in Brazil through matrix support since 2008. Matrix support service corresponds to an integrated care proposal and is a model of pedagogical-therapeutic intervention. It aims to produce and stimulate relationships among providers and patients, so as to foster information exchange and expand co-responsibility by the patient [5, 6].

However, although there have been significant contributions to the literature regarding facilitators and barriers to matrix support services [7, 8], little is known about the system’s quantitative impact on sharing cases between different levels of care [9]. A literature review regarding the pathways to mental Care in Brazil [10] showed that most patients who reach specialized mental health services are referred by non-Primary Care services, with hospitals and emergency services often being the first point of contact with the formal health network.

Additionally, continuity of care, especially regarding the return of patients from specialized services to Primary Care, is a well-known problem in Brazil [9, 11]. Thus, we hypothesized that a significant proportion of mental health patients treated in specialized services do not have their needs known by Primary Care. The aim of this study was to investigate the prevalence and factors associated with Primary Care registration of the mental health needs of patients treated at outpatient specialized services in a medium-sized city in Brazil with recent implementation of matrix support.

## Methods

### Design and participants

This is a document-based cross-sectional study conducted through an analysis of medical records of patients from outpatient specialized mental health services in a medium-sized city in Brazil. This city has approximately 120,000 inhabitants, is 80 km (50 mi) away from São Paulo, and has three specialized outpatient mental health services, namely: Psychosocial Care Center-II (PCC-II), Psychosocial Care Center for Psychoactive Substance Misuse (PCC-PSM), and the Outpatient Clinic.

A total of 413 patients were registered in the PCC-II, 403 patients were registered in the PCC-PSM, and 1142 patients were receiving mental health consultations at the Outpatient Clinic during the research period.

Inclusion criteria were being of adult age and having at least one registration for a mental health appointment between May 2018 and May 2019. Individuals were excluded if they resided in an area not covered by a Primary Care service that operates on the Family Health Strategy (FHS) model. Ultimately, 1198 patient records were included in this study.

### Data collection procedures

Data collection procedures were developed at the services between May and July 2019 and delivered by 16 trained individuals. These selected individuals were undergraduate students or undergraduate professionals in psychology or medicine and were supervised by 2 undergraduate professionals, one in nursing and one in social work. Data collectors filled out a form prepared for the purposes of the research when inclusion criteria were met based on data available in the medical records.

#### Primary care database

Before conducting this study, a database was created to compile Primary Care patients who had a record of complaints related to mental health. The inclusion of patients in this database considered the registration of at least one attendance due to mental health complaints with regard to section F of the International Statistical Classification of Diseases and Related Problems (ICD), or the registration of psychosomatic complaints associated with the use of a psychotropic medication belonging to group N of the WHO Anatomical Therapeutic Chemical Code (ATC) system. There is no electronic medical record in the municipality studied, only paper records are used. Thus, the entire patient record was accessed and read by the data collectors to track these records. Thus, 9834 patients among 60,411 patients covered by a Primary Care service operating on the FHS model were included in the database.

### Outcome

Our outcome was Primary Care registration of the mental health needs of patients treated at outpatient specialized mental health services. Therefore, all patients included in the study had their data cross-checked with data from the Primary Care database to assess this outcome. Patients were considered positive if they had a record of their mental health needs or a registration of psychosomatic complaints associated with the use of a psychotropic medication in both Primary Care and specialized services.

### Covariates

The following variables from the information included in medical records were considered of interest for this study: sex (male; female); age (18 to 30 years; 31 to 45 years; 46 to 60 years; 61+ years); diagnosis (neurotic disorders; affective disorders – unipolar; affective disorders – bipolar; psychosis; psychoactive substance misuse; intellectual disability or organic disorders; developmental disorders; no current diagnosis); referral source to the current service (spontaneous demand; Primary Care; emergency or hospital services; private services); and time attending the current service (up to 1 year; up to 3 years; up to 5 years; 5+ years).

### Statistical analysis

Statistical analyses were conducted with the use of the Stata 15 program (Stata Corporation, College Station, Texas USA). Descriptive statistical analysis with absolute and relative frequency estimates was performed for each of the covariates. There were no missing data for any of the studied variables.

Associations between the outcome (Primary Care registration of the mental health needs of patients treated at outpatient specialized services) and covariates were tested using unadjusted and adjusted logistic regression models. Adjustment took place in a single step and considered all included covariates.

### Ethical procedures

The study was submitted to and approved by the Ethics Committee of the School of Medical Sciences of the University of Campinas under opinion No. 3.065.312, following the Brazilian regulatory standards and guidelines for research involving human beings (CNS Resolution 466/2012). It was similarly in accordance with the provisions of the Declaration of Helsinki. The study fully ensured patients’ anonymity. Informed consent was obtained by all study participants. It adhered to the Guidelines for Strengthening the Reporting of Observational Studies in Epidemiology (STROBE Statement).

## Results

### Characterization of the participants

A total of 1198 patient records were included in this study, with 25% (*n* = 299) from Psychosocial Care Center II (PCC-II), 19.4% (*n* = 232) from the Psychosocial Care Center for Psychoactive Substance Misuse (PCC-PSM), and 55.7% from the Outpatient Clinic. The characterization of participants in relation to study covariates, by service and overall, is given in Table 1.

**Table 1:** Descriptive statistics of participants (*n* = 1198)

	PCC II		PCC-PSM		Outpatient Clinic		Overall	
	n	%	n	%	n	%	n	%
**Sex**								
Male	130	43.5	180	77.6	201	30.1	511	42.7
Female	169	56.5	52	22.4	466	69.9	687	57.3
**Age**								
18 to 30 years	50	16.7	86	37.1	167	25.1	303	25.3
31 to 45 years	98	32.8	74	31.9	152	22.8	324	27.1
46 to 60 years	104	34.8	64	27.6	205	30.7	373	31.1
61+ years	47	15.7	8	3.4	143	21.4	198	16.5
**Diagnosis**								
Neurotic disorders	33	11.0	9	3.9	271	40.7	313	26.1
Affective disorders (unipolar)	40	13.4	2	0.8	92	13.8	134	11.2
Affective disorders (bipolar)	52	17.4	5	2.2	12	1.8	69	5.8
Psychosis	121	40.5	4	1.7	25	3.7	150	12.5
Psychoactive substance misuse	8	2.7	74	31.9	17	2.5	99	8.3
Intellectual disability or Organic disorders	28	9.4	4	1.7	36	5.4	68	5.7
Developmental disorders	1	0.3	2	0.9	30	4.5	33	2.7
No current diagnosis	16	5.3	132	56.9	183	27.5	332	27.7
**Referral source for the current service**								
Spontaneous demand	47	15.7	117	50.4	434	65.1	598	49.9
Primary Care	64	21.4	29	12.5	205	30.7	298	24.9
Emergency or Hospital services	35	11.7	7	3.0	2	0.3	44	3.7
Other specialized service	141	47.2	19	8.2	7	1.1	167	13.9
Private Services	12	4.0	60	25.9	19	2.8	91	7.6
**Time attending the current service**								
Up to 1 year	62	20.7	136	58.6	223	33.4	421	35.2
Up to 3 years	52	17.4	30	12.9	161	24.1	243	20.3
Up to 5 years	48	16.1	21	9.1	262	39.3	331	27.6
5+ years	137	45.8	45	19.4	21	3.2	203	16.9
**TOTAL**	**299**	**100**	**232**	**100**	**667**	**100**	**1198**	**100**

PCC-II: Psychosocial Care Center-II; PCC-PSM: Psychosocial Care Center for Psychoactive Substance Misuse

### Primary care registration of patients’ needs

**Fig. 1:**
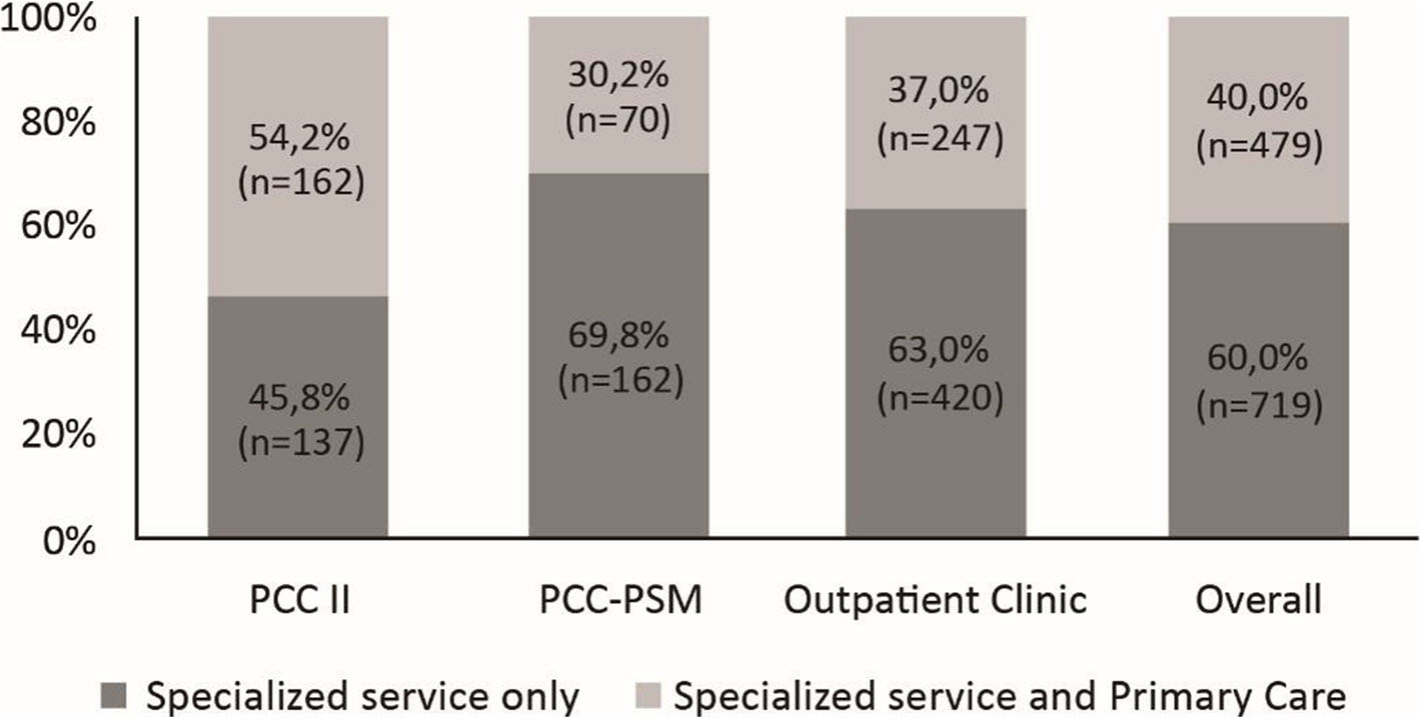
Prevalence of Primary Care registration of the mental health needs of patients treated at outpatient specialized services included in the study (n = 1198). Source: The authors, 2019. PCC-II: Psychosocial Care Center-II; PCC-PSM: Psychosocial Care Center for Psychoactive Substance Misuse

Overall, Primary Care registration of the mental health needs of patients treated at outpatient specialized services was calculated to be 40% (*n* = 479). There was evidence (*p* < 0.001) of significant differences in the prevalence of the outcome among different outpatient specialized services according to a chi-squared test (not in figure). The prevalence of users who had their mental health needs recorded in both specialized services and Primary Care services according to each studied service is shown in Fig. 1.

The prevalence of the outcome with regard to each of the covariates included in the study was investigated, as well as associations between covariates and the outcome using adjusted and unadjusted logistic regression. The results of these analyses are shown in Table 2.

**Table 2:** Unadjusted and adjusted^a^ associations between studied variables and Primary Care registration of the mental health needs are provided with the use of Logistic regression models. Odds Ratios (OR) with 95% corresponding confidence intervals (CIs) are presented (*n* = 1198)

	n	%	Unadjusted OR (95%CI)	Adjusted^**a**^ OR (95%CI)
**Sex**				
Male	511	37.2	1	1
Female	687	42.1	1.22 (0.97–1.55)	1.08 (0.82–1.42)
**Age**				
18 to 30	303	30.0	1	1
31 to 45	324	43.2	1.77 (1.27–2.46)	1.48 (1.03–2.10)
46 to 60	373	43.2	1.76 (1.28–2.43)	1.45 (1.02–2.07)
61 or more	198	43.9	1.82 (1.25–2.65)	1.56 (1.03–2.36)
**Diagnosis**				
Neurotic disorders	313	41.8	1	1
Affective disorders (unipolar)	134	44.8	1.12 (0.74–1.69)	0.94 (0.60–1.46)
Affective disorders (bipolar)	69	46.4	1.20 (0.71–2.02)	0.73 (0.40–1.33)
Psychosis	150	50.0	1.38 (0.93–2.05)	0.88 (0.53–1.43)
Psychoactive substance misuse	99	38.4	0.86 (0.54–1.37)	1.03 (0.59–1.80)
Intellectual disability or Organic disorders	68	45.6	1.16 (0.68–1.97)	0.95 (0.54–1.66)
Developmental disorders	33	21.1	0.37 (0.15–0.88)	0.51 (0.20–1.27)
No current diagnosis	332	31.6	0.64 (0.46–0.88)	0.78 (0.54–1.12)
**Current service**				
PCC-II	299	54.2	1	1
PCC-PSM	232	30.2	0.36 (0.25–0.52)	0.51 (0.30–0.87)
Outpatient Clinic	667	37.0	0.49 (0.37–0.65)	0.59 (0.37–0.92)
**Referral source to the current service**				
Spontaneous demand	598	35.3	1	1
Primary Care	298	43.6	1.41 (1.06–1.88)	1.30 (0.97–1.76)
Emergency or Hospital services	44	61.4	2.91 (1.55–5.46)	2.22 (1.11–4.45)
Other specialized service	167	51.5	1.94 (1.37–2.75)	1.26 (0.80–1.98)
Private Services	91	27.5	0.69 (0.42–1.13)	0.86 (0.50–1.48)
**Time attending the current service**				
Up to 1 year	421	34.9	1	1
Up to 3 years	243	42.8	1.39 (1.00–1.92)	1.20 (0.85–1.68)
Up to 5 years	331	39.6	1.22 (0.90–1.64)	1.02 (0.72–1.45)
5+ years	203	47.8	1.70 (1.21–2.39)	1.08 (0.71–1.63)

^a^: Adjusted for sex; age; diagnosis; current service; referral source to the current service; time attending the current service

Evidence was observed for an association between Primary Care registration of mental health needs and older patient age. All age groups over 31 years presented higher odds ratios for the outcome: 31 to 45 (OR: 1.48; 95%CI: 1.03, 2.10); 46 to 60 (OR: 1.45; 95%CI: 1.02, 2.07); 61+ (OR: 1.56; 95%CI: 1.03, 2.36).

There was no evidence for differences in the outcome by diagnosis after adjustment. However, we did observe an important difference related to the service that users were linked to. Namely, we found an inverse association with the outcome among PCC-PSM patients (OR: 0.51; 95%CI: 0.30, 0.87) and Outpatient Clinic patients (OR: 0.59; 95%CI: 0.37, 0.92) compared to PCC-II patients.

Finally, there was evidence for an association between referral by an emergency or hospital service to the current outpatient service, and Primary Care registration of mental health needs (OR: 2.22; 95%CI: 1.11, 4.45).

## Discussion

As far as we are aware, this is the first study to explore and attempt to quantify Primary Care registration of the mental health needs of patients treated at outpatient specialized mental health services. This study is particularly important in the Brazilian context given the functioning model of its mental health network. In contrast to Cuba, Spain and the United Kingdom, countries with a strong gatekeeping role by Primary Care and very low percentage of direct access (0–2.5%) [12], although the Brazilian health system works on the stepped care model, its specialized outpatient mental health services provide access by spontaneous demand.

Another development that should be highlighted in the Brazilian context is the recent reinsertion of the Outpatient Clinic as a component of the psychosocial care network by Ordinance No. 3588 (instituted December 21, 2017). As a result of psychiatric reform movements over four decades, the Brazilian health system had shifted to operating in a more favorable manner to community mental health services, such as Psychosocial Care Centers. However, due to political disputes in recent years, biomedical practice increasingly dominates mental health care [13]. This has been historically evidenced in Outpatient Clinics by the hierarchy among professionals and disarticulation among components of the mental health network [13, 14].

The above-mentioned information is relevant to understanding the results of the present study. It is noteworthy that the Outpatient Clinic and the PCC-PSM had the lowest prevalence of patients who had their mental health needs registered in Primary Care, while simultaneously demonstrating the highest percentage of patient intake by spontaneous demand.

Overall, Primary Care registration of the mental health needs of patients treated in specialized services was 40%, while the prevalence among PCC-II patients was 54.2%. In contrast, the Outpatient Clinic, rather than PCC-II, had the highest proportion of patients referred by Primary Care, but a prevalence of patient’s mental health needs registered in Primary Care of 37%. However, it should be noted that 47.2% of PCC-II patients were referred by other specialized services, mainly represented by the Outpatient Clinic. This scenario suggests a strongly centralized network at the Outpatient Clinic, which despite being a specialized service, seems to function as a gateway to mental Care in the studied municipality.

In comparison, the proportion of direct access (due to spontaneous demand) observed in our study, except for PCC-II, was higher than that found in countries such as Albania (40%), India (40%), Japan (39%) and Romania (39%), which are countries with the highest proportions of direct access among the 23 countries included in a review of pathways to mental Care worldwide [12].

Our criteria for determining the outcome included recording complaints related to mental health, a diagnosis report, or the use of psychotropic medications in the Primary Care records. Thus, it is assumed that some patients classified as negative for the outcome may have been assisted for other clinical conditions in Primary Care, even if their mental health treatment was not considered. Regardless, a scenario where only 40% of patients treated in specialized outpatient mental health services have their mental health needs registered in Primary Care is troubling. Integrated teams depend on the exchange of complete, current, up-to-date information to enable independent and collaborative work by members across various professions [15].

The National Comorbidity Survey Replication conducted in the United States of America found that 68% of adults with mental health disorders also face physical health problems [16]. Considering a similar scenario in our context of interest, a lack of awareness by Primary Care regarding mental health patients seen in specialized services may result in patients with mental disorders not having their clinical needs adequately addressed. Alternatively, such a lack of awareness may indicate that the clinical care performed in Primary Care does not consider the totality of the health needs of the patient.

As with Outpatient Clinic patients, PCC-PSM patients were less likely to have their mental health needs registered in Primary Care. It is noteworthy that care for users of psychoactive substances has historically been taboo in Primary Care, as care in Brazil is organized in a territorial manner [17, 18]. This is especially a problem for Community Health Agents (CHA) who are responsible for surveying patient needs [18]. Because they, by definition, live in the same community as their patients, these professionals do not wish to be mistaken as informants for the police or drug dealers [17]. Patients may themselves avoid seeking Primary Care for problems related to the use of psychoactive substances, although they occasionally access the service to obtain clinical health care [17, 19].

Another important factor is the growing presence of private services, such as Therapeutic Communities, as a component of the pathway to care for patients at the PCC-PSM. While a study conducted in Minas-Gerais, Brazil in 2016 [20] suggested a high prevalence of patients admitted through referral from other public services (44.7%), in addition to spontaneous demand (43.7%), our study identified a proportion of 25.9% of patients referred from private services. This is undoubtedly a topic that needs to be studied in greater depth. Therapeutic Communities promote an absence of the individual from society, are largely religious in nature, and many of them are not staffed by health professionals [21]. Thus, these services tend to use the equipment of the public health system, such as the PCC-PSM, to validate their treatments, but do not have the intention to integrate with other health network services, especially Primary Care.

In the current study, patients over 31 years of age were more likely to have their mental health needs registered in Primary Care. This is in line with data from previous studies on the use of Primary Care [22]. Older patients may be more in need of the care offered in Primary Care especially due to their higher incidence of chronic diseases [23].

We found strong evidence for an association between being referred by an emergency or hospital service and having mental health needs registered in Primary Care. These findings draw attention to important contradictions in the country’s health network. If, on the one hand, the proportion of referrals by hospital and emergency services was low when compared to those found in studies from the United Kingdom (28.9%) and Spain (24.3%) [12], on the other hand, it is important to consider that most of these referrals occurred because the patient went into a crisis, even after being attended by a Primary Care service. This points to the inability of Primary Care to manage or refer the case to a specialized service in a timely manner.

Alternatively, one cannot fail to consider the difficulties of Primary Care in referring cases to specialized services due to their high demand. It should be noted that patients who arrive at the service spontaneously in a moment of crisis are given priority intake, especially in the case of Psychosocial Care Centers, which operate in the open-door modality. Likewise, patients who have had a crisis recently stabilized by hospital and emergency services also have their referral prioritized. Thus, hindering the absorption of cases referred by Primary Care and favouring the creation of an alternative pathway to the specialized services.

Our results suggest that although the studied context has a matrix support service, it is still incipient and has not been effective in promoting sharing cases between different levels of care. There may be several factors that lead to the difficulty of operating this service. A literature review conducted previously [9] suggested that although it has been over 10 years since implementing matrix support in the Brazilian health system, it remains common for health networks to face problems such as: 1) lack of clarity in guidelines regarding sharing cases; 2) bureaucratization of the referral between services; 3) negative attitudes of professionals in dealing with mental health related issues; and 4) lack of institutionalized space and time for sharing of cases. It is noteworthy that these aspects are also highlighted in the study of international counterparts of this methodology, such as shared care [24].

Nonetheless, it is necessary to consider the important training gap in relation to mental health care experienced by both Primary Care and specialized Care professionals [9]. The proportion of hours devoted to the study of mental health care in undergraduate medical curricula in Brazil is still low. A study conducted in Goiás [25] estimated that this proportion was 1.2% of the total hours of the course. A national review of undergraduate courses [26] concluded that the hours allocated to studying mental health care in the field of nursing corresponded to 2.4% in private institutions and 3.5% in public institutions.

Thus, it is necessary to create strategies to address this scenario. In addition to strengthening matrix support teams, bottom-up change should be implemented in order to establish a setting which promotes action by professionals. One important strategy may be to raise awareness and promote training of Primary Care professionals, especially CHA, in identifying cases in a timely manner. It is also crucial for specialized services to improve the counter-referral of patients for Primary Care, thereby improving attainment of patient care and facilitating return of the patient from specialized to Primary Care.

Some limitations should be considered when interpreting our results. First, the incompleteness of data in the medical records limited including information which could be relevant to studying the current outcome, such as ethnicity, income, paid work status, information on screening chronic diseases, and markers of social support, among others. The lack of this information brings to light the idea proposed by Saraceno [27] about the existence of “strong variables” and “shadow variables” in constituting elements which play a prominent role in evaluating the evolution of a mental disorder. According to the author, the strong variables are related to the diagnosis, condition severity and disease history, while the shadow variables are those related to the individual resources, such as intellectual capacity, degree of information, etc. Saraceno [27] also argues that these last variables would be left in the “shadows” because they are considered irrelevant with regard to the evolution of the disease; however, a previous Brazilian study has already shown that sociodemographic variables such as education and having health insurance influence the receipt of mental health care and the pathway taken by the patients [28].

Second, patients classified as negative for the outcome may have been assisted for other clinical conditions in Primary Care, even if their mental health condition was not considered. Alternatively, it is necessary to consider the possibility that Primary Care professionals may be aware of the treatment of their patients in outpatient specialized services in some cases, but that this was not reflected in the coded data due to lack of documentation.

Nevertheless, our study provides valuable information for reorienting practice regarding sharing cases in both the studied context and in other locations with similar arrangements. This information can be an important starting point for proposing implementation studies which seek to assess the impact of integration strategies to qualify aspects such as acceptability, adoption, penetration, and fidelity of matrix support initiatives.

## Conclusions

In conclusion, we found evidence to support our hypothesis that a significant proportion of mental health patients seen in specialized services do not have their needs known by Primary Care. We observed associations of the outcome with patient age, and with referral source; namely, if patients were referred by an emergency or hospital service. At the same time, we observed an inverse association between the outcome and the patient’s affiliation with the Outpatient Clinic or the Psychosocial Care Center for psychoactive substance misuse, suggesting important differences among the studied services and their relationship to the Care network.

Our study provides relevant information for contexts where access to specialized mental health services occurs in different ways. The results demonstrate that even with the provision of mechanisms for network integration, such as matrix support, groundwork is necessary to ensure that sharing cases between Primary Care and specialized services is effective. We highlight the need to raise awareness among and improve the training of Primary Care professionals, as well as improve the counter-referral of patients from specialized services to Primary Care.

Our study also provides information on the proportion of patients with mental disorders who may not be receiving clinical care, contributing to worsen health disparities experienced by this population, especially with regard to psychotic patients and users of psychoactive substances.

## Data Availability

The datasets generated and analysed during the current study are not publicly available due to ethical concerns regarding privacy and confidentiality of participants, but are available from the corresponding author upon reasonable request.

## Abbreviations

ATC: Anatomical Therapeutic Chemical Code
CHA: Community Health Agents
FHS: Family Health Strategy
ICD: International Classification of Diseases
PCC-II: Psychosocial Care Center II
PCC-PSM: Psychosocial Care Center for Psychoactive Substance Misuse
